# Readmission Following Perioperative Myocardial Injury: Clinical Predictors and Impact on Mortality

**DOI:** 10.1155/2022/7674962

**Published:** 2022-08-13

**Authors:** Alex Anzelmi, Yasser Khalil, Martin E. Matsumura

**Affiliations:** The Pearsall Heart Hospital, Geisinger Wyoming Valley Hospital, Wilkes-Barre 18711, PA, USA

## Abstract

**Background:**

Perioperative myocardial injury (PMI) following noncardiac surgery is associated with a high risk for mortality, and readmission within 30 days of PMI increases this risk. Identifying risk factors for readmission among survivors of PMI is critical to improving outcomes in PMI. We examined risk factors for readmission following discharge after surgery complicated by PMI and the effect of readmission on 1-year mortality.

**Methods:**

The study is a retropective cohort analysis of patients diagnosed with PMI in a single health system over a 10-year period. Univariate predictors of readmission were used to construct a multivariable logistic regression model. Mortality was assessed using Kaplan–Meyer survival analysis.

**Results:**

Of the 207,729 surgical patients, 5159 (2.5%) had PMI. By 30 days following PMI, 1254 patients (24.3%) died, 1142 (22.2%) were readmitted but alive at 30 days, and 2763 patients (53.5%) were alive and had not been readmitted. Readmitted patients were older, had higher peak troponin levels, and were more likely to have prior coronary, neoplastic, lung, and kidney disease. Multivariable logistic regression revealed increasing age and peak troponin, prior cancer diagnosis, and chronic lung and kidney disease as independent predictors of readmission. Readmitted patients had higher 1-year mortality than those not readmitted (33.9% vs. 22.2%, *p* < 0.001).

**Conclusions:**

Readmission following PMI is associated with increased mortality in the following year. Patients suffering from PMI who are at risk of readmission are older, have a greater extent of myocardial injury, and are more likely to have chronic comorbidities. Identification of patients at risk of readmission following PMI is critical to improving both outcomes and utilization of hospital resources.

## 1. Introduction

Perioperative acute myocardial infarction (AMI) following noncardiac surgery is associated with a very high risk of both in-hospital and 30-day mortality [1–5]. Studies of perioperative patients meeting the definition of AMI, specifically elevated levels of cardiac troponin accompanied by symptoms and/or electrocardiographic changes of ischemia, suggest perioperative MI occurs in up to 5% of noncardiac surgeries [1–3]. The concept of perioperative myocardial injury (PMI), also described as myocardial injury following noncardiac surgery (MINS), has been increasingly accepted as a clinically important complication occurring after noncardiac surgery [6–10]. As opposed to perioperative AMI, PMI is diagnosed on the basis of elevated biomarkers of cardiac injury following surgery, without accompanying findings of AMI, including symptoms and electrocardiographic indicators of myocardial ischemia [6–9, 11]. PMI occurs in 10–20% of unselected postoperative patients, and a growing body of literature has demonstrated an up to fourfold increase in short-term and long-term mortality in this patient population [12].

30-day hospital readmission is a commonly used quality metric given the association between hospital readmission and increased cost of care and adverse patient outcomes [13, 14]. Focus on 30-day readmission rates among surgical patients has increased following the introduction of the Hospital Readmissions Reduction Program which introduced financial penalties tied to hospital readmission rates for select diagnoses/procedures [14, 15]. In addition, noncardiac surgery patients are included in publicly reported readmission measures by the Center for Medicare and Medicaid Services under all-cause readmissions [16]. In addition to increased mortality both during index hospitalization and postdischarge, patients experiencing perioperative AMI are also at significantly increased risk of 30-day hospital readmission [3]. Not surprising, patients who experience perioperative AMI and require 30-day readmission have poor outcomes, with a recent large retrospective analysis demonstrating in-hospital and 6-month mortality of 11.3% and 17.6%, respectively. [3].

Understanding patient characteristics that are associated with hospital readmission following PMI offers an opportunity to better understand the course of PMI, more effectively identify patients at risk for poor outcomes, and target these individuals for closer follow-up and enhanced management strategies. Unfortunately, there is currently a paucity of data regarding which, if any, patient characteristics are risk factors for readmission following PMI. In the present study, we evaluated the risk factors associated with early (30 day) mortality and hospital readmission of patients suffering from PMI over a 10-year period in a single-integrated health system. In addition, we evaluated the prognostic importance of readmission on 1-year mortality in this population. Our goal was to gain a better understanding of which PMI patients are at risk for hospital readmission in order to develop a strategy to intervene in both readmission and the high mortality rate associated with PMI.

## 2. Methods

The present study was a retrospective review of all patients ≥18 years of age who underwent elective, urgent, and emergent noncardiac surgery in the Geisinger Health System between 2007 and 2016. Patients were identified from the Geisinger electronic medical record and were included in the present study if they had cardiac troponin *T* (cTnT) measurements performed with rising and falling kinetics and at least 2 values greater than the 99^th^ percentile within the first 72 hours following surgery. [17] Those patients with elevated cTnT were designated as AMI if they had this diagnosis coded based on abnormal cTnT associated with criteria for AMI; otherwise they were designated as PMI. [17] Patients undergoing ambulatory procedures were excluded. Data on patient demographics and results of echocardiographic assessment of the left ventricular ejection fraction and performance of coronary angiography were collected from the medical record. Comorbid diagnoses were pulled from ICD-10 diagnoses of each patient's medical record.

Patients who were readmitted within 30 days of discharge from the index surgery were identified from the medical record. These patients were included in the readmission group unless the readmission was a previously scheduled elective readmission. Mortality was determined for the first year after the index surgical admission by review of the medical record.

Comparisons between PMI patient groups who were or were not readmitted within 30 days were made using Student's *t*-test or the rank sum test for continuous variables and chi-squared analysis for categorical variables. Significant variables associated with readmission were included in a multivariable logistic regression model to determine independent predictors of readmission. Survival to 1 year of patients readmitted within 30 days than those not readmitted was assessed by Kaplan–Meier survival analysis. For all comparisons, *p* < 0.05 was considered significant. Statistical analyses were performed with Sigmastat v4.0 software (Systat, San Jose CA). The study was approved by the Institutional Review Board of Geisinger Health System.

## 3. Results

**Figure 1:**
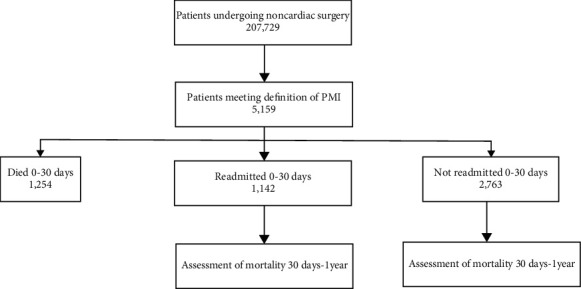
Schematic of patient breakdown. Patients meeting definition of PMI (see methods) were assigned to (a) died discharge to 30 days, (b) readmitted within 30 days, (c) not readmitted within 30 days. Groups (b) and (c) were followed for up to 1 year from index discharge.

A total of 207,729 unique noncardiac surgical procedures were performed in the Geisinger Health System from 2007 through 2016, of which 5159 were complicated by documented PMI (2.5%).  Figure 1 summarizes patient assignments and outcomes. By 30 days following the index surgical admission, 1254 patients (24.3%) with PMI died, 1142 (22.2%) had an unscheduled hospital readmission but were alive at 30 days, and 2763 (53.5%) were alive and had not had a readmission.

Characteristics of patients with PMI as a whole and comparison of those who died or were readmitted within 30 days of surgery than those alive/not readmitted are summarized in Table 1. Patients who died or were readmitted were older and had a greater degree of myocardial injury as defined by peak cTnT. In addition, these patients had significantly higher background of diabetes mellitus, chronic kidney disease, coronary artery disease, prior stroke, prior cancer diagnosis, and chronic lung disease compared to patients not readmitted following PMI. There were significantly more patients who underwent coronary angiography in the group who died or were readmitted, although this was a very small percentage of the total cohort (0.9%). In contrast, there was no significant difference in percentages of PMI patients who were diagnosed with AMI between the 2 groups.

A multivariable regression model was constructed from significant univariate predictors of 30-day death and readmission (Table 2). The following factors were independently associated with 30-day mortality or readmission (odd ratio, 5%–95%, *p* value): increasing age (1.013, 1.009–1.018, *p* < 0.001), peak cTnT (1.028, 1.012–1.044, *p* < 0.001), prior cancer diagnosis (1.152, 1.017–1.304, *p*=0.026), and chronic lung (1.169, 1.032–1.325, *p*=0.014) and kidney disease (1.584, 1.395–1.797, *p* < 0.001). Due to the very small number of patients who underwent cardiac catheterization, this variable was not included in the multivariate model.

**Figure 2:**
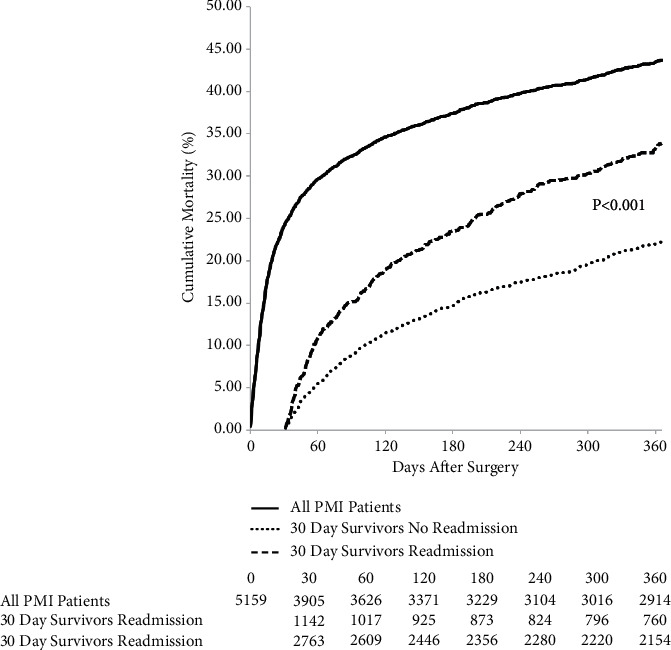
Mortality to 1 year following index surgical discharge. Solid line= overall cumulative mortality including index hospitalization to 30 days after discharge; dashed line= cumulative mortality of the 30-day readmission cohort from 30 days to 1 year; Dotted line= cumulative mortality of the nonreadmitted cohort from 30 days to 1 year.

Mortality beyond 30 days to 1 year following surgery was further analyzed after stratification by the occurrence of 30-day readmission (Figure 2). Within 30 days of discharge, 1254 (24.3%) died. Among 30-day survivors, readmission within the first 30 postoperative days remained a significant predictor of longer term mortality up to 1 year after index surgery with 33.9% of readmitted patients dead at 1 year compared to 22.2% of patients not readmitted (154% increase in mortality, *p* < 0.001).

## 4. Discussion

Patients suffering from perioperative ischemic events meeting the definition of PMI following noncardiac surgery are at a high risk of morbidity and mortality [6–9]. However, incidence of and risk factors for 30-day hospital readmission, a metric which is increasingly recognized as important as it relates to both quality and overall cost of care, has yet to be studied in this patient population. [13] In the present study, we evaluated the factors associated with 30-day hospital readmission and mortality among patients with PMI. We found that increasing patient age and infarct size as defined by peak cTnT, as well as preexistent cancer, lung disease, and renal disease, were independent predictors of readmission following PMI. To the best of our knowledge, this is the first study examining the specific risk factors for mortality and readmission among a large cohort of patients suffering from PMI. In addition, we found that 30-day readmission identifies patients at a very high risk of mortality at 1 year in patients following PMI, which runs parallel to the findings of prior studies on perioperative AMI patients. [3] These data suggest that readmission within 30 days of PMI is associated with substantial subsequent mortality risk and that the aforementioned factors may help identify these very high-risk individuals early in their initial hospital course. While the association of PMI and mortality does not clearly imply causation, it is reasonable to conclude that PMI puts patients at significant risk of all-cause mortality in the year following surgery. Identification of patients at a risk of readmission may help to direct postdischarge resources toward those patients and lead to improvement of both readmission rates and poor short-term and long-term outcomes.

It is notable that our analysis of PMI was based on an abnormal cTnT value drawn on selected patients following surgery. In contrast to prior studies that assessed troponin in all patients in the perioperative period, the cTnT assessments in the present study were based on clinical suspicion of myocardial ischemia by the perioperative care team. Recently, it has become clear that the majority of patients who have perioperative cardiac injury as defined by elevation of myocardial specific cardiac troponins do not have symptoms or clinical findings meeting the universal definition of AMI [18, 19]. For this reason, it is likely that the actual incidence of PMI was underrepresented in our database. In support of this concept, in our study, 2.5% of patients had PMI, compared to 11–17% of patients in studies that relied on routine postoperative troponin surveillance. Interestingly, other studies have suggested that mortality after PMI is independent of whether elevated troponin is in isolation or in association with findings fulfilling the definition of MI [10, 20]. Similarly, in the present study, we found that the percentage of PMI patients who fulfilled criteria for AMI was not different between the cohorts with and without 30-day death or readmission.

As previously mentioned, a prior study demonstrated increased 6-month mortality in patients suffering from perioperative cardiac events followed by readmission [7]. In contrast to the present study, that study used ICD-9 codes for the diagnosis of myocardial infarction. As a result, only 0.2% of patients in that study were diagnosed with perioperative AMI, which likely resulted in a different risk profile than our study. Despite that, similar to the present study, that study noted that nearly a third of patients diagnosed with perioperative MI died or were readmitted in the first 30 days, compared to the present study in which 46.5% of PMI patients died or were readmitted in the first month following hospital discharge.

The low rate (<1%) of cardiac catheterization in our cohort warrants consideration. A prior study noted performance of cardiac catheterization in a significantly higher percentage of PMI patients (20.8%) compared to 0.9% in the present study. [1] Of note, 21% of the patients in that study had ST elevation myocardial infarctions, a finding that would likely drive the use of cardiac catheterization. Because of the low utilization of cardiac catheterization in our patients, it is impossible to comment on the predominance of cardiac (i.e., Type I or II myocardial infarction) vs. extracardiac (i.e., sepsis, pulmonary embolus, and so on) PMI among our study population. [21].

It is important to acknowledge the limitations of the present study and how these limitations affect interpretation of the data. As previously noted, the study is retrospective, and therefore, the indications for assessment of and clinical scenario associated with troponin elevations in the PMI patients were not assessed. The fact that assessment of cardiac troponins was based on clinical suspicion is an important shortcoming: it allows the possibility that elevated troponins were simply a marker of severe medical or hemodynamic instability in these patients, and therefore, PMI was not the driver but rather a marker of readmission risk and poorer outcomes in these patients. [22, 23] We did not define the cause of mortality among patients who died up to year postoperative, and given the high prevalence of significant comorbidities such as cancer and kidney disease, it is likely that a significant proportion of deaths were unrelated to either the index surgery or associated PMI. In addition, we did not examine the association of the type of surgery with mortality. It is likely that high-risk scenarios such as emergent surgeries were associated with both PMI and mortality, without clear cause and effect between the 2 events. [10] Lastly, the contemporary use of high sensitivity troponin assays to identify PMI may significantly change the utility of routine detection of perioperative ischemia, and the results of the current study may not be applicable to these new assays. [24].

## 5. Conclusion

In summary, the present study identifies risk factors for 30-day hospital readmission following PMI and confirms prior findings of an increased 1-year mortality risk following PMI. Patients suffering from PMI have strikingly high rates of in-hospital and short-term and long-term mortality. Initiatives aimed at the identification of patients at the highest risk of mortality among those who suffer from PMI are critical to defining interventions to improving outcomes in this very high-risk population. PMI patients with significant risk factors for poor outcomes should be the target of focused intervention aimed at preventing adverse events in the year following surgical admission.

## Figures and Tables

**Table 1:** Characteristics of PMI patients overall and comparison of PMI patients grouped by 30-day death/readmission +/−.

	All PMI patients *n* = 5159	PMI 30-day death/readmission (+) *n* = 2396	PMI 30-day death/readmission (−) *n* = 2763	*p*
Age, median (range)	72 (18–100)	73 (19−100)	70 (18−100)	<0.001
Male (%)	2862 (55.5)	1354 (56.5)	1508 (54.6)	0.180
BMI, kg/M^2^, mean (std dev)	29.7 (8.24)	29.5 (7.65)	30.0 (8.44)	0.070
Peak TnT, ng/dL, mean (range)	0.908 (0.04–95.83)	1.13 (0.04-81.09)	0.715 (0.04−95.83)	<0.001
Criteria for perioperative myocardial infarction (%)	841 (16.3)	364 (15.2)	477 (17.3)	0.057
Ejection fraction, median (range)	55 (10−75)	55 (10−65)	55 (20−75)	0.113
Cardiac catheterization (%)	48 (0.9)	44 (1.8)	4 (0.2)	<0.001
Smoker (%)	3081 (59.7)	1449 (60.5)	1632 (59.1)	0.320
Diabetes (%)	2023 (39.2)	983 (41.0)	1040 (37.6)	0.014
Renal disease (%)	2156 (41.8)	1080 (45.1)	1076 (38.9)	<0.001
Coronary artery disease (%)	2255 (43.7)	1094 (45.7)	1161 (42.0)	0.008
Prior myocardial infarction	1435 (27.8)	689 (28.8)	746 (27.0)	0.159
Prior stroke (%)	658 (12.8)	330 (13.8)	328 (11.9)	0.046
Hypertension (%)	3266 (63.3)	1527 (63.7)	1739 (62.9)	0.572
Hyperlipidemia (%)	2857 (55.4)	1331(55.6)	1526 (55.2)	0.795
Chronic lung disease	1567 (30.4)	788 (32.9)	779 (28.2)	<0.001
Prior cancer diagnosis	1448 (28.1)	728 (30.4)	720 (26.1)	<0.001
Peripheral arterial disease	1141 (22.1)	547 (22.8)	594 (21.5)	0.276

Continuous variables were compared using the Mann–Whitney *U*-test (age and ejection fraction) or Student's *t*-test (BMI and peak TnT). Categorical variables were compared using the Pearson chi-square test. BMI = body mass index; TnT = troponin T.

**Table 2:** Results of the multivariable logistic regression analysis model based on univariate predictors of 30-day death/readmission.

	Odd Ratio (95% CI)	*p*
Age	1.013 (1.009−1.018)	<0.001
Peak troponin T	1.028 (1.012−1.044)	<0.001
Diabetes	1.081 (0.954−1.224)	0222
Renal disease	1.584 (1.395−1.797)	<0.001
Coronary artery disease	1.051 (0.940−1.077)	0.429
Prior stroke	1.089 (0.921−1.287)	0.320
Prior cancer	1.152 (1.017−1.304)	0.026
Lung disease	1.169 (1.032−1.325)	0.014

## Data Availability

Data will be made available upon request.

## Abbreviations

AMI: Acute myocardial infarction
PMI: Perioperative myocardial injury
MINS: Myocardial infarction following non-cardiac surgery
cTnT: Cardiac troponin T.
